# Morphological Analysis of Elastic Fibers in Various Grades of Oral Squamous Cell Carcinoma and Epithelial Dysplasia Using Verhoeff–Van Gieson Stain

**DOI:** 10.5041/RMMJ.10367

**Published:** 2019-07-18

**Authors:** Janardhanam Dineshshankar, Nalliappan Ganapathy, Thuckanaickenpalayam Ragunathan Yoithapprabhunath, Jeyaraman Swathiraman, Thangadurai Maheswaran, Vadivel Ilayaraja

**Affiliations:** Department of Oral Pathology and Microbiology, Vivekanandha Dental College for Women, Elayampalayam, Tiruchengode, Namakkal District, Tamilnadu, India

**Keywords:** Elastic fibers, oral squamous cell carcinoma, Verhoeff–Van Gieson stain

## Abstract

**Background:**

Oral squamous cell carcinoma (OSCC) is the sixth most common malignancy in India. The aggressiveness of OSCC is analyzed not only based on the dysplastic features and tumor infiltration pattern, but also by means of the stromal changes that pave the way for an invasion into the connective tissue. The role of elastic fibers in the progression of OSCC is still unknown because of sparse literature and the masking effect of overlying inflammatory cells and the lower number of elastic fibers in the lamina propria. The present study provides further insight into the qualitative assessment of elastic fibers in various grades of dysplasia and OSCC.

**Objectives:**

To analyze the morphological changes exhibited by the elastic fibers in epithelial dysplasia and OSCC.

**Materials and methods:**

Two sections were cut from each of 60 samples of varying grades of OSCC and 60 samples of varying grades of epithelial dysplasia followed by staining with hematoxylin and eosin and Verhoeff–Van Gieson stain.

**Results:**

Statistically significant results were obtained for qualitative analysis of elastic fibers. A change in density and orientation to overlying epithelium and tumor islands was seen on progressing from well-differentiated to poorly differentiated OSCC and in progressing grades of dysplasia.

**Conclusion:**

The uniqueness of this study lies in the exploration of elastic fibers in dysplasia and well-differentiated OSCC, a less explored field. The study of the connective tissue stromal changes can be used as an adjunct to histological grading.

## INTRODUCION

Oral cancer remains a deadly disease even though many advances have been made in treatment modalities and therapeutic approaches over the recent decades. Among the oral cancers, oral squamous cell carcinoma is the most common malignant epithelial neoplasm affecting the oral cavity, constituting about 94% of all oral malignancies. It is the sixth leading cancer by incidence worldwide.^1^

Oral squamous cell carcinoma includes two components: the tumor epithelial cells and the connective tissue stroma.^2^ The stroma is an essential component for the maintenance of epithelium, and it acts as a scaffold for cellular adhesion.^3^ When the epithelium changes, so does the stroma.^4^ The reactive changes in the tumor stroma may alter the biological aggressiveness of oral cancer.^5^ These stromal changes and the invasion of tumor cells into the stroma lead to metastasis, which is the hallmark of malignancy.^3^ The interaction between the epithelial dysplastic cells and the stromal cells leads to invasion. This interaction is essential for the development of malignancy. The aggressiveness of oral squamous cell carcinoma is not only based on the dysplastic features and tumor infiltration pattern but is also due to the stromal changes that pave the way for an invasion into the connective tissue. Oral potentially malignant disorders (PMD) have an increased risk of progressing to cancer. The most common PMD is leukoplakia; the histopathological findings in leukoplakia vary from hyperkeratosis without epithelial dysplasia to various degrees of epithelial dysplasia and even carcinoma *in situ*, frank squamous cell carcinoma, and verrucous carcinoma.^6^,^7^ Assessment and prognosis of transformation potential depends on the histologic grading, which is done by quantifying the degree of architectural and cellular changes.^8^ As epithelial dysplasia and oral squamous cell carcinoma are epithelial lesions, only the epithelial component has been included in the grading system, but the connective tissue component is often ignored while grading dysplasia and squamous cell carcinoma. By incorporating these stromal changes into the grading system, it helps in the better prediction of the course and the outcome of the disease. The epithelial component has been studied widely, while the connective tissue component is an unexplored area until now.^2^ Among the stromal components the collagen fiber has been studied extensively by various authors, whereas other stromal components have not so far been studied.

Elastic fibers that are the principal components of connective tissue next to collagen fibers are composed of proteins like elastin, elaunin, and oxytalan. According to Mecham and Davis (1994) elastic fibers develop by the deposition of tropoelastin, which is the precursor of mature elastin.^9^ The elastic fibers present in various connective tissues are responsible for physiologic elasticity of the organs. These fibers consist of two distinct components: elastin and elastic fiber microfibrils.^10^ As per the literature, there is communication between the tumor cells and elastic fibers, but its effect on the progression of tumor is uncertain.^2^

Hence, the current study was instigated with an aim to evaluate the morphological changes exhibited by the elastic fibers which is one of the principal components of the connective tissue in varying grades of oral squamous cell carcinoma (OSCC) and epithelial dysplasia.

## MATERIALS AND METHODS

This study was performed using a total of 120 formalin-fixed paraffin-embedded soft-tissue samples of varying grades of OSCC and epithelial dysplasia which were retrieved from the archives of the department of oral pathology and microbiology, Vivekanandha Dental College for Women, Tiruchengode, during 2015–2017 after obtaining institutional ethical committee clearance. We investigated 20 blocks in each group of OSCC (20 each in well-differentiated OSCC, moderately differentiated OSCC, and poorly differentiated OSCC) and 20 blocks in epithelial dysplasia (20 each from mild, moderate, and severe dysplasia). Two sections of 4 μm thickness were made from each block; one section of each sample was stained with hematoxylin and eosin under the standard protocol for confirmation of diagnosis, and the other section with Verhoeff–van Gieson stain. The stained slides were observed under a light microscope. Two independent observers evaluated all the slides to reduce subjective bias. Samples without adequate connective tissue thickness were excluded from the study. Statistical analysis was done by using chi-square test.

### Qualitative Analysis of the Elastic Fibers

The elastic fibers were analyzed at 10× and 40× magnifications for density, morphology, pattern of grouping, staining intensity, and orientation of elastic fibers to the overlying epithelium and to the tumor islands. The density was categorized as scanty, moderate, or abundant. The morphology and pattern of grouping was categorized as long, thin, straight elastic fibers, and present singly, or short, thick, wavy elastic fibers, present in bunches. The staining intensity was categorized as weak, moderate, or strong. Categorization of the orientation of elastic fibers to the overlying epithelium was parallel or perpendicular, and orientation to the tumor islands was parallel or haphazard.

## RESULTS

**Figure 1:**
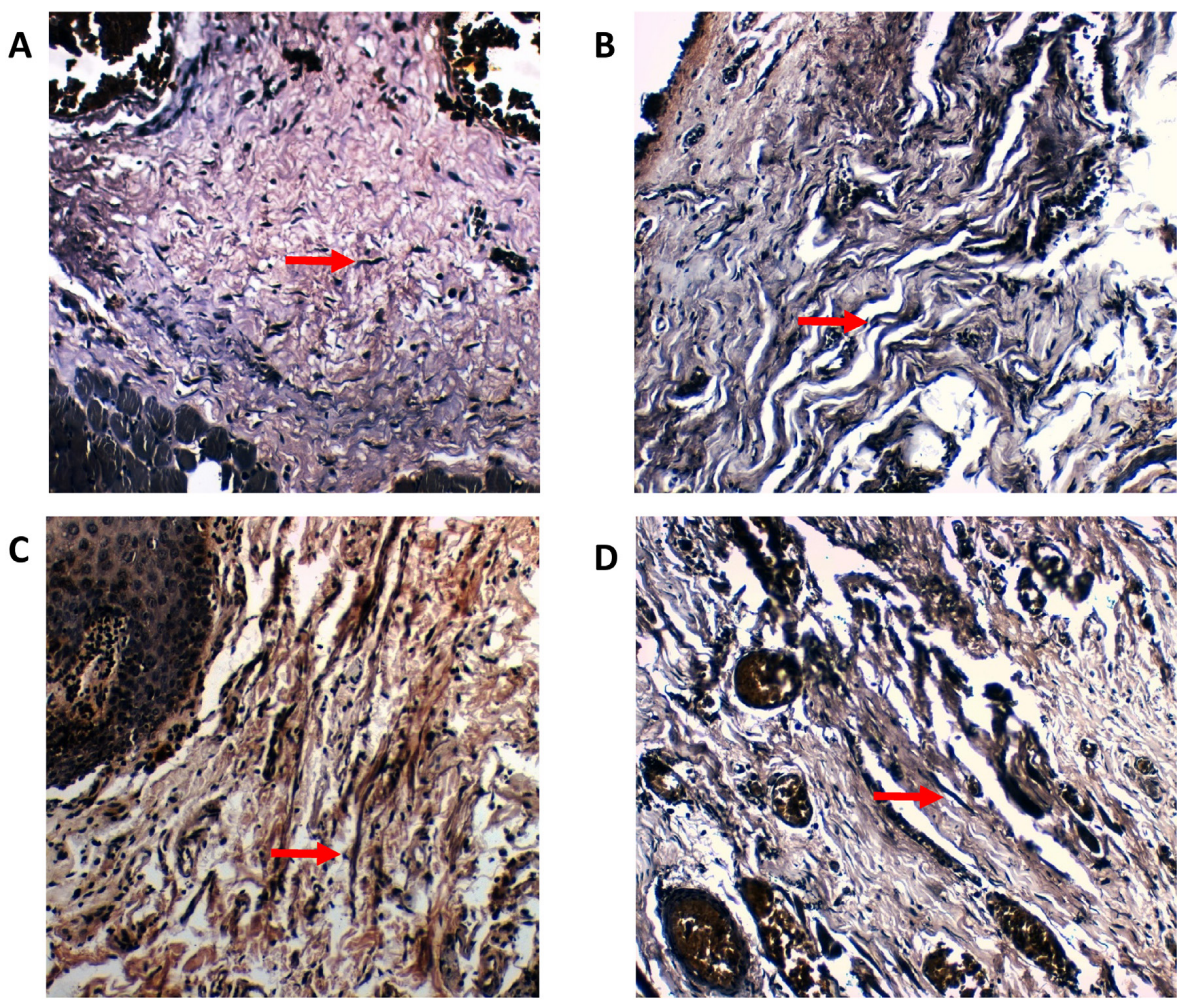
Histopathological Images Showing Verhoeff–van Gieson-stained Elastic Fibers. **A:** Arrow indicates short, thick, wavy fibers in well-differentiated OSCC.**B:** Arrow indicates short, thick, wavy fibers in mild dysplasia. **C:** Arrow indicates long, thin, straight fibers in poorly differentiated OSCC. **D:** Arrow indicates long, thin, straight fibers in severe dysplasia. All panels are shown at ×20 magnification.

When the density of the elastic fibers was analyzed, scanty fibers were noted in well differentiated OSCC and poorly differentiated OSCC) and abundant fibers were noted in moderately differentiated OSCC; whereas in epithelial dysplasia the density was moderate in mild and moderate dysplasia cases, and scanty in severe dysplasia. The evaluation of morphology noted short, thick, wavy fibers in both well differentiated OSCC (Figure 1A) and moderately differentiated OSCC and in mild dysplasia (Figure 1B) and moderate dysplasia; whereas as the grades progress long, thin, straight fibers were seen in poorly differentiated OSCC (Figure 1C) and in severe dysplasia (Figure 1D). The pattern of grouping indicates that the fibers were present singly in all the grades of OSCC and in moderate and severe dysplasia, whereas in mild dysplasia the fibers were seen in bunches. The staining intensity was weak in mild dysplasia, moderate in well-differentiated OSCC, poorly differentiated OSCC and in moderate and severe dysplasia, and strong in moderately differentiated OSCC. The orientation of elastic fibers to the overlying epithelium changed from parallel in well-differentiated OSCC, moderately differentiated OSCC, and mild and moderate dysplasia, to a perpendicular orientation in poorly differentiated OSCC and in severe dysplasia. The analysis of orientation of elastic fibers relative to tumor islands revealed that the orientation changed from parallel in well-differentiated OSCC to a haphazard form in moderately differentiated OSCC and poorly differentiated OSCC (Table 1).

**Table 1:** Elastic Fiber Parameters for Varying Grades of Oral Squamous Cell Carcinoma (OSCC) and Epithelial Dysplasia.

Fiber Parameter	Chi-square (*P* value)	Mild Dysplasia (*n*=20)	Moderate Dysplasia (*n*=20)	Severe Dysplasia (*n*=20)	Well-diff. OSCC (*n*=20)	Moderately Diff. OSCC (*n*=20)	Poorly Diff. OSCC (*n*=20)
Elastic fiber density	114.33 (<0.001)						
Scanty		–	4	20	12	–	20

Moderate		18	16	–	8	6	–

Abundant		2	-	6	-	14	–

Morphology	120.00 (<0.001)						
Short, thick, wavy		20	20	-	20	20	–

Long, thin, straight		-	-	20	-	-	20

Grouping pattern	10.17 (0.071)						
Single		18	20	20	20	20	20

In bunches		2	–	**-**	-	**-**	**-**

Staining intensity	93.9 (<0.001)						
Weak		14	2	2	2	–	2

Moderate		2	12	16	16	4	16

Strong		4	6	2	2	16	2

Orientation to overlying epithelium	75.16 (<0.001)						
Parallel		20	20	–	16	14	4

Perpendicular		–	–	20	4	6	16

Orientation to tumor islands	20.04 (<0.001)						
Parallel		NA	NA	NA	18	6	4

Haphazard		NA	NA	NA	2	14	16

## DISCUSSION

Carcinomas are malignant epithelial tumors in which epithelial cells illustrate an unusual arrangement with unreliable degrees of differentiation. Epithelial tumor cells invade the stroma in groups or alveoli, which are implanted in or bounded by an extracellular matrix (ECM) producing reactive changes in the stroma. The ECM molecules influence differentiation, proliferation, and migration of epithelial tumor cells. The reactive alteration in the cancer stroma may modify the biological aggressiveness of oral cancer.^11^

The ECM has an effect on tumor behavior; improper synthesis or degradation of any ECM molecule can change cell functioning and thus helps in the progression of disease. Matrix metalloproteinases (MMPs) produced by the cancer-associated fibroblasts as well as the inflammatory cells degrade the ECM structural components, both collagen and elastic, and thus help in neoplastic progression.^12^

Investigation on the association between the complex dysplastic grading system and the stromal reaction in oral lesions is incomplete,^13^,^14^ and the majority of the studies focus on epithelial cells.^15^,^16^ The role of elastic fibers in the evolution of OSCC and epithelial dysplasia is still unknown.^2^

The current study seeks to provide new knowledge based on the qualitative analysis of elastic fibers in varying grades of OSCC and epithelial dysplasia. To the best of our knowledge, there is no published research that compares both OSCC and epithelial dysplasia in the English literature to shed light on possible alterations in the elastic fibers. The uniqueness of this study lies in the exploration of elastic fibers in various grades of OSCC and epithelial dysplasia, which is still a less explored arena.

As per the literature, there is communication between tumor cells and elastic fibers, but its outcome on the progression of carcinoma is questionable.^2^ Lapis and Timar have stated that tumor cell–elastin interactions have biological significance in tumor progression.^17^ In addition, small elastin degradation products with soluble elastin itself were shown to be chemotactic for cancer cells.^18^

George et al. conducted a study on OSCC and observed elastic fibers along with many other components of tumor stroma. They only mentioned that the amount of elastin in the stroma of OSCC was less as compared to collagen, and no details were given regarding the pattern and morphology of elastic fibers in various grades of OSCC.^11^ The study by Ma described a primary increase followed by a decrease in the amount of elastic fibers with increasing grades of carcinoma.^19^ In contrast, a study performed by Zhang et al. revealed a decrease in elastic fibers with neoplastic progression from epithelial atypia to early invasive carcinoma.^20^

Our study revealed a statistically significant change in the density of the elastic fibers that varied from scanty in well differentiated OSCC to abundant in moderately differentiated and poorly differentiated OSCC. In dysplasia cases, the fiber density was moderate in mild and moderate dysplasia and scanty in severe dysplasia. The evaluation of morphology noted short, thick, wavy fibers in both well and moderately differentiated OSCC, and in mild and moderate dysplasia. As the grades progress, long, thin, straight fibers were seen in poorly differentiated OSCC and in severe dysplasia. In line with our study, Kardam et al.^2^ observed a similar change in OSCC regarding the morphology of elastic fibers. The pattern of grouping indicates that the fibers were present singly in all the grades of OSCC and in moderate and severe dysplasia, whereas in mild dysplasia the fibers were seen in bunches. We also included a new parameter, namely staining intensity, which was weak in mild dysplasia, moderate in well differentiated and poorly differentiated OSCC and in moderate and severe dysplasia, and strong in moderately differentiated OSCC. In contrast to the study by Kardam et al.,^2^ in our study the assessment of orientation to the overlying epithelium showed a statistically significant difference among various grades of OSCC and dysplasia. As the grade progresses, orientation to overlying epithelium changed from parallel to perpendicular. Orientation of the elastic fibers relative to the tumor islands also changed.

## CONCLUSION

Stromal change may alter the biological behavior or the aggressiveness of the tumor. Elastic fibers are showing different patterns in various grades of OSCC and epithelial dysplasia. Thus, the stromal changes can be used as an adjunct in histological grading. There is a need to carry out further detailed studies on stromal changes occurring during the progression of a tumor.

## Abbreviations

ECM: extracellular matrix
MMPs: matrix metalloproteinases
OSCC: oral squamous cell carcinoma
PMD: potentially malignant disorders.
